# Exendin-4 Ameliorates Traumatic Brain Injury-Induced Cognitive Impairment in Rats

**DOI:** 10.1371/journal.pone.0082016

**Published:** 2013-12-02

**Authors:** Katharine Eakin, Yazhou Li, Yung-Hsiao Chiang, Barry J. Hoffer, Hilary Rosenheim, Nigel H. Greig, Jonathan P. Miller

**Affiliations:** 1 Department of Neurosurgery, Case Western Reserve University School of Medicine, Cleveland, Ohio, United States of America; 2 Drug Design and Development Section, Laboratory of Translational Gerontology, Intramural Research Program, National Institute on Aging, NIH, Baltimore, Maryland, United States of America; 3 Ph.D. Program for Neural Regenerative Medicine, Graduate Institute of Neural Regenerative Medicine, Taipei Medical University, Taipei City, Taiwan (R.O.C.); 4 Department of Neurosurgery, Taipei Medical University Hospital, Taipei City, Taiwan (R.O.C.); University of South Florida, United States of America

## Abstract

Traumatic brain injury represents a major public health issue that affects 1.7 million Americans each year and is a primary contributing factor (30.5%) of all injury-related deaths in the United States. The occurrence of traumatic brain injury is likely underestimated and thus has been termed “a silent epidemic”. Exendin-4 is a long-acting glucagon-like peptide-1 receptor agonist approved for the treatment of type 2 diabetes mellitus that not only effectively induces glucose-dependent insulin secretion to regulate blood glucose levels but also reduces apoptotic cell death of pancreatic β-cells. Accumulating evidence also supports a neurotrophic and neuroprotective role of glucagon-like peptide-1 in an array of cellular and animal neurodegeneration models. In this study, we evaluated the neuroprotective effects of Exendin-4 using a glutamate toxicity model *in vitro* and fluid percussion injury *in vivo*. We found neuroprotective effects of Exendin-4 both *in vitro*, using markers of cell death, and *in vivo*, using markers of cognitive function, as assessed by Morris Water Maze. In combination with the reported benefits of ex-4 in other TBI models, these data support repositioning of Exendin-4 as a potential treatment for traumatic brain injury.

## Introduction

Traumatic brain injury (TBI) represents a major public health issue that affects 1.7 million Americans each year and is a primary contributing factor (30.5%) of all injury-related deaths in the United States [1,2]. The occurrence of TBI is likely underestimated and thus has been termed “a silent epidemic” [3,4]. Mild to moderate TBI account for 80 to 95% of cases, with severe TBI forming the remainder [5]. The elderly, in particular, are vulnerable to TBI and suffer an increased mortality and worse functional outcome in the face of lower initial injury severity [6].Many survivors experience prolonged or even permanent neurocognitive dysfunction, with lasting changes in cognition, motor function, and personality [7,8]. A conservative estimate is that 3.2 million Americans, or 1.5% of the population, currently live with long-term disabilities after TBI, and these disabilities are estimated to cost $9.2 billion in lifetime medical costs and $51.2 billion in productivity losses [9]. 

The pathophysiology of TBI can be divided into primary and secondary injury processes. Primary injury refers to injury at the time of trauma caused by direct impact or penetrating injury. Secondary injury denotes a cascade of molecular mechanisms that are initiated at the time of trauma and evolve in the hours and days after the traumatic event. These mechanisms include glutamatergic excitotoxicity, free-radical injury to cell membranes, electrolyte imbalances, mitochondrial dysfunction, neuroinflammatory responses, apoptosis, and secondary ischemia from vasospasm [10–14]. Since these processes are believed to be responsible for the progressive neurological impairment after TBI, the development of effective therapeutic strategies capable of arresting secondary injury-induced damage has become a focus of intense research activity over the last two decades, both in the clinical and preclinical settings, in light of a current lack of an effective pharmacological treatment for TBI.

Exendin-4 (Ex-4) is a long-acting glucagon-like peptide-1 (GLP-1) agonist approved for the treatment of type 2 diabetes mellitus (T2DM) that not only effectively induces glucose-dependent insulin secretion to regulate blood glucose levels but also reduces apoptotic cell death of pancreatic β-cells [15,16].In addition to its presence in the pancreas, the GLP-1 receptor (GLP-1R) is expressed on neurons throughout the brain, which GLP-1 and Ex-4 readily enters [17]. Accumulating evidence from *in vitro* and *in vivo* models of apoptosis, neurodegeneration, and brain injury show that GLP-1 receptor activation leads to a variety of neurotrophic and neuroprotective effects [18]. Brain transplantation of GLP-1 secreting mesenchymal stem cells has been shown to reduce cellular pathology after traumatic brain injury [19]. One of the mechanisms for these protective effects is the activation of protein kinase A (PKA) and phosphoinositide 3-kinase (PI3K) signaling pathways, which are involved in cell survival and metabolism [20–23]. Pre-treatment of cultured rat hippocampal neurons with Ex-4 prevented Aβ- and iron-induced cell death [20] and, in addition, SH-SY5Y human neuroblastoma cells were protected from hydrogen peroxide-induced apoptosis [24]. Ex-4 also protected against glutamate-mediated excitotoxicity in primary hippocampal neurons by modulating the resulting pathological increase in intracellular calcium concentration [25,26], which is a key contributor to secondary injury effects following TBI and other brain injuries such as stroke [26].

Recent studies have shown that Ex-4 is effective in mitigating cognitive impairments after mild TBI in a mouse weight-drop model [27] and prevented injury-induced changes in gene expression, including genes and pathways associated with oxidative stress, neuroinflammation, and neurodegenerative diseases such as Alzheimer’s disease [22,28]. However, the therapeutic benefit of Ex-4-treatment following moderate-severity diffuse TBI has not been addressed. In the present study, we evaluated the therapeutic potential of the GLP-1R agonist, Ex-4 in terms of: (1) its protection against glutamate excitotoxicity in rat primary hippocampal neurons and human SH-SY5Y cells, and (2) its ability to minimize TBI-induced cognitive impairment after moderate diffuse TBI in the rat. No single animal model of TBI perfectly mimics the human condition and activity across different TBI models provides further support for translational relevance [29,30].

## Materials and Methods

### In vitro studies

Human SH-SY5Y neuroblastoma cells known to express the GLP-1R [31] were cultured in Eagle's Minimum Essential Medium and Ham's F12 Medium (1:1 mix), supplemented with 10% heat-inactivated fetal bovine serum (FCS) and 100 U/mL penicillin/streptomycin (Invitrogen, Carlsbad, CA). Cultures were grown in a humidified incubator (5% CO2 and 95% air) at 37°C. Medium was replaced every other day and cells were subdivided (1:3 ratio) every 5 days (0.25% trypsin, 0.53 mM EDTA solution) or on reaching 80% confluence.

To obtain rat primary neurons, the hippocampal area was obtained from day 18 Sprague-Dawley rats and subjected to mild trypsinization [25]. Cells then were seeded onto either polyethyleneimine-coated plastic dishes or 22 mm^2^ glass cover slips, and were incubated in neurobasal medium containing B-27 supplements, 2 mM L-glutamine and 1 mM HEPES (Life Technologies, Gaithersburg, MD) together with 0.001% gentamic in sulfate (Sigma-Aldrich, St Louis, MO). Greater than 90% of the resulting cells presented classical neuron-like morphology. All studies were undertaken within 10 days of plating.

For verification of GLP-1R expression, total RNA was extracted from both SH-SY5Y cells and rat cerebral cortical neurons (at day 10 in culture) using Trizol (Invitrogen, Carlsbad, CA). Cells were combined from 10 wells, and RNA quantity and quality were validated by spectrophotometer at wavelengths 260 and 280 nMλ. To degrade genomic DNA, RNA (1 μg) was treated with DNase I (Ambion Inc.) and a 50 ng sample assessed for each one-step RT-PCR reaction (QIAGEN One Step RT-PCR Kit). The primers for human SH-SY5Y neuroblastoma cells were: human GLP-1R forward: 5’ TCAAGGTCAACGGCTTATTAG 3’ and reverse: R: 5’ TAACGTGTCCCTAGATGAACC 3’ [31], showing the predicted PCR product of 480 bp. Secondary primers for human GLP-1R primers were: forward 5’ TTCTGCAACCGGACC 3’ and reverse 5’ CAAGTGCTCAAGCCG 3’, with an expected product size is 1.1kb. For rat primary neurons primers were: rat GLP-1R, forward: 5’ AGTAGTGTGCTCCAAGGGCAT 3’ and reverse: 5’ AAGAAAGTGCGTACCCCACCG 3’, displaying the anticipated PCR product of 190 bp; for rat GAPDH, forward: 5’ GACCTGCAGAGCTCCAATCAAC 3’ and reverse: 5’ CACGACCCTCAGTACCAAAGGG 3’, showing the expected PCR product of 214 bp. For a positive control, RNA was extracted from a line of CHO cells permanently transfected with rat GLP-1R. RT-PCR conditions were: 50°C for 30 min; 95°C for 15 min followed by 35 cycles of 95°C for 30 s, 56°C for 30 s, 72°C for 30 s; then 72°C for 10 min. 

Glutamate toxicity is implicated as a key mechanism underlying neuronal dysfunction and death following TBI [32,33]. Hence, SH-SY5Y neuronal cultures were challenged with excess glutamate to evaluate Ex-4 mediated neuroprotective actions. A dose-response analysis was initially performed to define a glutamate concentration and exposure time to provide significant yet incomplete cellular death. From these preliminary studies, cultures were either pre-treated for 1 hr with Ex-4 and then challenged with 100 mM glutamate or were co-challenged with glutamate followed rapidly by Ex-4. For SH-SY5Y cells viability was assessed by MTS assay (Promega, Madison, WI) at 24 hr following glutamate challenge and caspase-3 activity was quantified in a parallel series of cells at 18 hr by a colorimetric caspase-3 assay kit (Sigma Aldrich, St. Louis, MO), as per the manufacturer’s protocol. Primary neurons were challenged with 10 uM glutamate and apoptosis was assessed by staining with Hoechst 33342 (Invitrogen). Briefly, following fixation in paraformaldehyde (4%), Triton X-100 (0.2%) was added to permeabilize cell membranes. Incubation with Hoechst 33342 (1 μM for 30 min) allowed visualization of nuclei by fluorescence microscopy. The percent of cells possessing condensed or fragmented nuclei, and hence considered apoptotic, was evaluated in fields containing 200 cells at a minimum.

### Rodent in vivo studies

Male Sprague-Dawley rats (Harlan Laboratories Inc., Indianapolis, IN) weighing 350-400 grams were used in the present study. Animals were housed under a 12hr light/dark cycle, provided with food and water *ad libitum*, and were randomly assigned to either sham (*n* = 9), TBI (*n* = 9), or TBI+Ex-4 (*n* = 8) groups. All animal procedures were conducted in accordance with NIH guidelines reviewed and approved by the Institutional Animal Care and Use Committee (IACUC) of Case Western Reserve University (Protocol #2011-0610). The number of animals used was the minimum number based on power calculations (α=0.05 and 1-ß=0.80). All surgery was done under aseptic conditions and postoperative analgesia was provided by administration of NSAIDs. We could not use opiates postoperatively as they have been shown to be neuroprotective which would confound interpretation of the results.

The fluid-percussion injury (FPI) device used to produce experimental TBI was identical to that described by others [34]. All rats were surgically prepared for midline FPI (mFPI). Briefly, animals were anesthetized using 4% isoflurane gas, a 4.8 mm diameter burr hole was produced midline between the coronal and lambdoid sutures, and a Leur-Loc hub was affixed to the perimeter of the burr hole using cyanoacrylate. Dental acrylic and two small nickel-plated screws were used to anchor the hub to the skull. Twenty-four hours later, at the time of injury, the rats were anesthetized, the surgical site exposed, and the animal was connected to the injury device. The force of the injury administered was between 1.86 to 2.04 atmospheres of pressure (atm) and resulted in the suppression of the righting reflex for a period between 6 and 9 min. Sham animals were connected to the injury device but no injury was delivered and suppression of the righting reflex lasted less than 60 sec. 

For drug administration, Ex-4 (Bachem, Torrance, CA) was dissolved in 0.9% sterile saline and administered via a subcutaneously placed Alzet mini-osmotic pump (model 2001, DURECT Corporation, Cupertino, CA) for a period of 7 consecutive days at the rate of 3.5 pM/kg/min (equivalent to 21.1 µg/kg/day). This dose was based on prior studies that demonstrated positive effects on gene expression and improved novel object recognition in a mouse mild TBI weight-drop injury model [27,28]. This dose of Ex-4 was also associated with neuroprotective effects against oxidative stress, apoptosis, and trophic factor withdrawal in mouse models of neurodegeneration [35,36]. The Alzet pumps were filled, primed for 6 hr in sterile 0.9% saline, and were then implanted 30 min post-injury (subcutaneously posterior to the scapulae) under isoflurane anesthesia. A loading dose of Ex-4 (2.52 nM/kg i.p. equivalent to the amount of drug released by the pump over 12 hours) was administered at the start of the implantation surgery to obtain immediate therapeutic levels of the drug upon placement of the Alzet pump. Seven days after implantation, the implanted pumps were removed under isoflurane anesthesia using aseptic technique and the surgical site was sutured aseptically. 

For cognitive assessment, spatial learning and memory were assessed using the Morris water maze (MWM) task, as detailed elsewhere [37]. Briefly, a circular tank (180 cm in diameter, 45 cm in height) was filled with water maintained between 25-28°C. Maze performance and video tracking for each animal was measured using EthoVision XT 8.5 (Noldus Information Technology Inc., Leesburg, VA). The goal of the task was to locate a hidden submerged platform that is 15 cm in diameter and located 2 cm below the surface of the water. The location of the platform was fixed across all the trials during hidden platform testing. Distinct visual cues on the walls of the maze room, which remained constant across all trials, provided spatial cues to assist animals in locating the hidden platform. Rats were tested for four trials per day over four days. In each trial, the rat was placed into the water from one of four cardinal directions in a pseudo-random order and allowed up to 120 s to locate the platform. Animals unable to locate the platform within 120 s were gently guided to it by the experimenter. All animals were allowed to remain on the platform for 30 s before being removed from the tank and placed in a heated incubator for 10 min until the next trial. A probe trial was performed after 24 hr following completion of the hidden platform testing. This probe trial consisted of a single 30s trial, with the platform removed. Briefly, each animal was placed in the maze and allowed to swim freely for 30 s. The number of platform zone crossings, defined as the diameter of the platform plus an additional 7.5 cm radius surrounding the border of the platform, was recorded.

Statistical analyses were performed using the Statistical Package for the Social Sciences (SPSS, Inc., Chicago, IL). Two- tailed Student’s t-test and analysis of variance (ANOVA) was used for parametric data, and Fisher’s exact test was used for categorical data. In cases when more than a single group was compared to the control group, a Dunnetts test was applied. A p value of 0.05 or less was defined as statistically significant, as detailed within the Figure legends.

## Results

**Figure 1:**
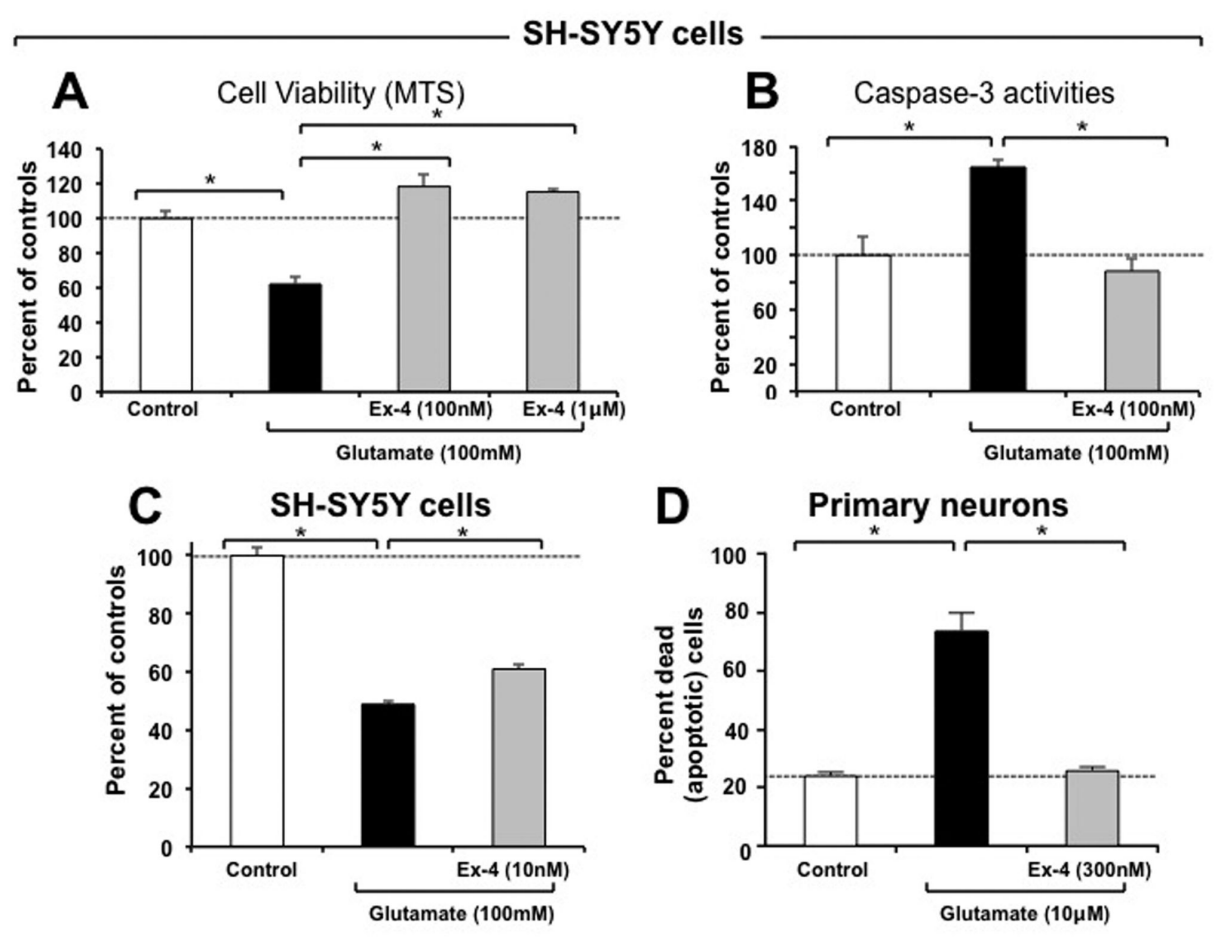
Studies of Exendin-4 in vitro. Activation of GLP-1R signaling by Exendin-4 (Ex-4) protects both human SH-SY5Y cells and rat primary neurons from glutamate-induced excitotoxicity. (**A**) The cellular viability of SH-SY5Y cells challenged with glutamate (100 mM) was significantly reduced (37.5%) following 24 hr incubation, as assessed by MTS assay. Pretreatment with Ex-4 (100 nM) fully mitigated this glutamate-induced cellular loss (p<0.05 *vs*. glutamate alone challenged group, Dunnett’s *t*-test, N≥3 per treatment group). (**B**) In line with glutamate-induced excitotoxic cell loss, caspase-3 levels were significantly elevated in SH-SY5Y cells. Pretreatment with Exendin-4 fully ameliorated this rise (p<0.05 *vs*. glutamate alone challenged group, Dunnett’s T test, N≥3 per treatment group). (**C**) Co-treatment of SH-SY5Y cells with glutamate (100 mM) and concentrations as low as 10 nM Ex-4 resulted in significant mitigation of excitotoxicity, as assessed by MTS assay at 24 hr (p<0.05 *vs*. glutamate alone challenged group, Dunnett’s *t*-test, N≥3 per treatment group). (**D**) In parallel studies, rat primary neurons proved vulnerable to glutamate challenge (10 μM) which induced substantial apoptosis (73.1%). Pretreatment with Ex-4 (300 nM) significantly mitigated this, lowering levels to those of unchallenged control neuronal cultures (p<0.05 *vs*. glutamate alone challenged group, Dunnett’s *t*-test, N≥3 per treatment group). The glutamate challenge concentrations (100 mM for SH-SY5Y cells and 10 μM for primary neurons) and time line were selected from preliminary studies focused on inducing a significant yet incomplete cellular loss to allow for assessment of mitigation as well as a potential intensification of cell death by the experimental treatment.

Human SH-SY5Y cells and rat primary neurons express the GLP-1R whose activation by Exendin-4 is protective against glutamate-induced excitotoxicity. The presence of the GLP-1R was verified, in accord with prior studies [21,36], on both SH-SY5Y human neuroblastoma cells and rat primary neurons by RT-RCR; GLP-1R mRNA possessed the expected RT–PCR product size of 190 bp. As SH-SY5Y and rat primary neuron cell lines have been widely used in the study of neuroprotective mechanisms, they were challenged with glutamate toxicity in the presence and absence of Ex-4. As illustrated in Figure 1A, challenge with glutamate (100 mM) reduced SH-SY5Y cell viability by 37.5% (p<0.05 vs. control), which was fully ameliorated by Ex-4 (100 nM and 1uM, p<0.05%). The glutamate-induced loss in cell viability was accompanied by a dramatic rise in caspase-3 activity (164%, p<0.05 vs. control) that was blocked by Ex-4 (Figure 1B). As pretreatment of neurons in living brain would be difficult to accomplish prior to a TBI incident, in separate studies SH-SY5Y cells were challenged with a similar dose of glutamate followed quickly by Ex-4. As illustrated in Figure 1C, glutamate-induced excitotoxicity was mitigated by Ex-4 at a dose of 10 nM, lowering cellular loss from 51% to 39%. Rat primary neurons showed a greater vulnerability to glutamate excitotoxicity, demonstrating a loss parallel to SH-SY5Y challenged cells at a glutamate concentration of 10 uM (not shown). In line with this loss of cell viability, there was an increase in the percent of primary neurons undergoing apoptosis in the presence of glutamate (10 uM), which rose from 23.8% in controls to 73.1% (Figure 1D). This elevation was completely blocked by Ex-4. 

**Figure 2:**
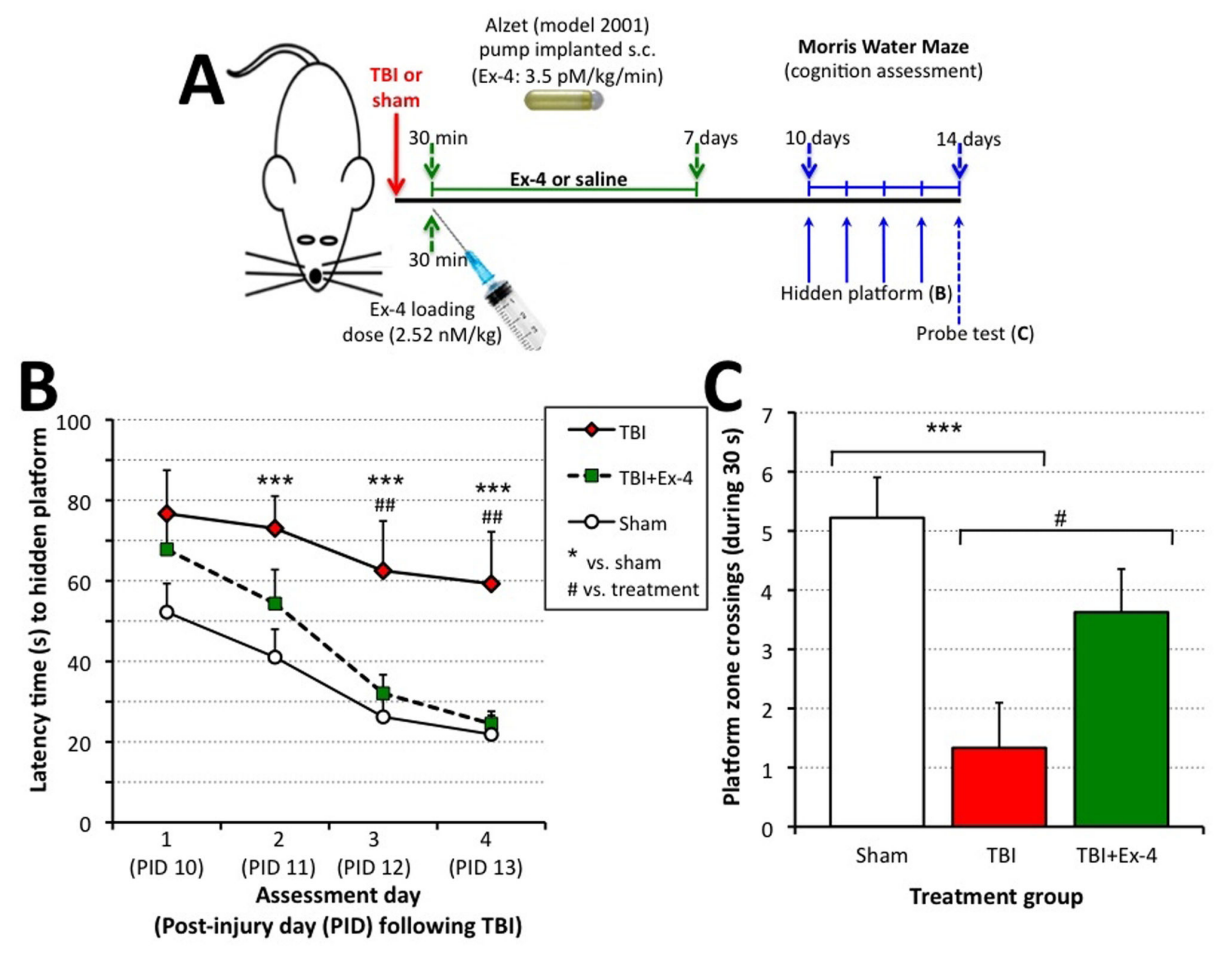
Studies of Exendin-4 in vivo. Administration of Exendin-4 (Ex-4) significantly ameliorated cognitive impairments induced by fluid percussion-induced TBI in rats assessed by the Morris Water Maze (MWM) paradigm. (**A**) Time line of study: 30 min following fluid percussion injury-induced TBI or sham treatment, rats were administered either saline or Ex-4 for 7 days (initially, a loading dose of Ex-4 was administered followed by steady-state subcutaneous (s.c.) infusion via an implanted Alzet mini pump). Thereafter on days 10 to 14, animals were subjected to MWM assessment. (**B**). Post-injury administration of Ex-4 significantly improved MWM performance. Cognitive performance as assessed by latency to locate the hidden platform in the MWM was compared for each of the three treatment groups, TBI (*n* = 9), TBI+Ex-4 (*n* = 8), sham (*n* = 9). A repeated-measures ANOVA showed significant between group differences. Post hoc analysis using Fishers LSD revealed that animals that were treated with Ex-4 after TBI had significantly shorter latencies to reach the goal platform as compared to injured saline-treated. No significant difference was observed in the performance between TBI+Ex-4 and sham groups. Data are presented as the mean ± SEM. ***p≤ .001, sham relative to TBI. ***^††^*** *p*≤ .01, Ex-4 relative to TBI. (**C**) Number of platform zone crossings during the probe trial. Cumulative learning for the platform location was assessed during the probe trial as the number of times the animals swam within a 7.5 cm radius of the platform border (i.e., 2x the diameter of the platform). One-way ANOVA indicated significant between group differences. Fisher’s LSD post hoc showed that sham and TBI+Ex-4 had better retention of the platform location relative to animals that received TBI only. Data are presented as the mean ± SEM. Brackets indicate direct comparisons between groups. *p≤ .05, ***p ≤ .001.

Rats administered Exendin-4 therapy following moderate TBI were cognitively unimpaired, as assessed by Morris Water Maze. In light of recent studies demonstrating amelioration of cognitive impairments in mice subjected to concussive mild TBI [27,38] and to assess translation across TBI models, rats were subjected to moderate midline FPI or sham injury and spatial learning and memory were evaluated using the Morris water maze (MWM) (Figure 2A). Testing was performed on post injury days (PID) 10 to 14. Hidden platform testing was performed over four days corresponding to PID 10 through 13. Cognitive performance (i.e., latency to reach the goal platform) was analyzed using a repeated-measures ANOVA and revealed a significant between-group difference, *F*(2, 24) = 9.176, *p* < .01 (Figure 2B). A Fisher LSD post hoc test revealed that the TBI group had significantly longer escape latencies (i.e., worse performance) as compared to the TBI+Ex-4 and sham groups, *p* < .01 and < .001 respectively. There was no significant difference in the latency to reach the goal platform between TBI+Ex-4 and Sham groups, *p* = .354.

The probe trial consisted of a single 30 sec trial that was performed on PID 14, after the final day of hidden platform testing. During this test the platform was removed, the animal was allowed to swim freely for 30 sec, and the number of platform zone crossings (defined as 2x the diameter of the platform) was analyzed across groups. These data are presented in Figure 2C. A one-way ANOVA revealed a significant difference in the mean number of platform crossings between the groups, *F*(2,23) = 7.496, *p* < .01. Fisher LSD post hoc analysis showed significant differences between the sham and TBI groups, *p* < .01. Animals in the sham and TBI+Ex-4 groups had significantly more platform zone crossings relative to the TBI group, *p* < .01 and < .05, respectively. There was no difference between the TBI+Ex-4 and sham groups, *p* = .138 (i.e. not statistically significantly different). Swim times to a raised and visible platform were similar, indicating little effect on swim speed or visual acuity after FPI.

## Discussion

In this study we examined the therapeutic efficacy of Ex-4 in preventing glutamate-induced toxicity in neuronal cell cultures, as an indicator of its potential to modify secondary injury effects. In addition, we evaluated the role of Ex-4 treatment in ameliorating TBI-induced cognitive deficits as assessed by the MWM. 

Similar to what has been reported in the literature, we confirmed the presence of the GLP-1R in both human and rat cell cultures [24,31]. We then evaluated the role of Ex-4 in preventing glutamate-induced cell death. Glutamate-induced reductions in cell viability and increased caspase-3 activity were blocked by pre-treatment with Ex-4 in SH-SY5Y cells and rat primary hippocampal neurons at concentrations found to be effective in mitigating cell death in other cellular studies [21,22,26]. The effect of Ex-4 treatment in the present study replicates our findings from previous studies across a number of models [18,25] and a number of insults, including oxidative stress, hypoxia, trophic factor withdrawal and toxic malformed proteins (such as amyloid-β peptide) [18,20-22,24-27].

Glutamate excitotoxicity is a key element involved in the secondary injury cascade that is initiated immediately following the primary insult. Treatment with Ex-4 prior to exposure to pathological glutamate levels in cell culture completely blocks apoptosis. Based on the literature, this effect is likely due to the action of Ex-4 on the class B family of 7-transmembrane-spanning, heterotrimeric G-protein-coupled receptors. Receptor activation stimulates adenylyl cyclase via the G-protein Gα; this in turn causes an increase in cAMP levels that regulates many diverse cellular responses including the activity of pro-survival factors [18,31,39]. Ex-4 increases cAMP in cell culture and pharmacologically-mediated increases in cAMP were found to reduce cognitive deficits following TBI [40].

FPI is the most commonly used rodent model of TBI because it accurately replicates many of the cognitive and histological changes seen in human head injury [34]. Midline FPI produces diffuse brain injury that has been shown to produce hippocampal-dependent impairment of cognitive function in the absence of overt cell death [41].The hippocampal formation underlies many functions related to cognition, especially memory formation, and is known to be selectively vulnerable in human TBI. Clinical and experimental observations suggest that memory-related cognitive processes are disproportionately affected by TBI-induced pathology, and the severity of these deficits correlate well with level of injury, suggesting that functional changes in these structures underlie much of the pathology related to TBI [42,43].

The Morris Water Maze is a test of spatial learning and memory that assesses the ability of a rodent to encode and retrieve spatial memory for the location of a hidden submerged platform [37,44,45]. The MWM has been extensively utilized in TBI; not only is it among the most sensitive tests for TBI, but the extent of dysfunction in the MWM has been shown to correlate well with injury severity, suggesting that it is an appropriate test to assess TBI-induced injury and recovery [46–49].

The Ex-4 dose (3.5 pM/kg/min equivalent to 21 µg/kg/day) used here has not only proven efficacious in both AD and amyotrophic lateral sclerosis preclinical mouse models [35,36], but also compares favorably with Ex-4 dosing in human type 2 diabetes mellitus (once weekly exenatide LAR: 2 mg/week administration delivers 5.7 μg/kg/day for a 50 kg person; [50]). 

We previously reported that pre-treatment with Ex-4 initiated 48 h prior to mild TBI allowed the development of steady-state plasma and brain levels of drug in a mouse weight-drop injury model, and completely reversed injury-induced deficits in cognitive performance in a novel object recognition task [14] event. Hypothermia has been shown to be neuroprotective in a number of different brain injury protocols. We examined this potential confound in a recent study [38] and found no significant effect of this dose of Ex-4 on body temperature.

The GLP-1R is expressed in several regions of rodent and human brain areas, supporting the use of CNS mediated GLP-1 receptor agonists for treatment of human neurological disorders [20,51–53]. The efficacy of GLP-1 receptor signaling observed here is in line with descriptions of brain entry of GLP-1 and Ex-4 [17], and GLP-1 receptor-mediated actions in various CNS neurological conditions (for review see, 18,54–56). Since GLP-1 receptor stimulation is effective in multiple disease models including AD, PD, HD, ALS, stroke and peripheral neuropathy, it is likely that there are common mechanisms in the later stages of neuronal cell death observed in these conditions [20–22,35,57,58].

No single model of TBI perfectly mimics the human condition; therefore, it is important to evaluate potential therapies across different TBI models, which provides support for translational relevance [29,30]. The findings from the present study show that Ex-4 prevents cell death in vitro and ameliorates TBI-induced cognitive deficits in vivo. Obviously, one cannot directly extrapolate mechanism from in vitro studies to in vivo behavioral effects. Thus, the changes in MWM behavior cannot be stated to be due to glutamate toxicity, based on our data. These data, along with the accumulating evidence in support of the beneficial effects of Ex-4 in other animal models of TBI, suggest that Ex-4 may be a valuable therapeutic agent for the treatment of human TBI.
